# Anti-biofilm efficacy of green-synthesized ZnO nanoparticles on oral biofilm: *In vitro* and *in silico* study

**DOI:** 10.3389/fmicb.2022.939390

**Published:** 2022-10-03

**Authors:** Dibyajit Lahiri, Rina Rani Ray, Tanmay Sarkar, Vijay Jagdish Upadhye, Sujay Ghosh, Soumya Pandit, Siddhartha Pati, Hisham Atan Edinur, Zulhisyam Abdul Kari, Moupriya Nag, Muhammad Rajaei Ahmad Mohd Zain

**Affiliations:** ^1^Department of Biotechnology, University of Engineering & Management Kolkata, Kolkata, India; ^2^Department of Biotechnology, Maulana Abul Kalam Azad University of Technology, Haringhata, West Bengal, India; ^3^Department of Food Processing Technology, Malda Polytechnic, West Bengal State Council of Technical Education, Government of West Bengal, Malda, India; ^4^Parul University, Vadodara, Gujrat, India; ^5^AMH Energy Pvt. Ltd., Kolkata, India; ^6^Department of Biotechnology, Sharda University, Noida, India; ^7^Natnov Bioscience Private Limited, Balasore, India; ^8^Skills Innovation & Academic Network (SIAN) Institute, Association for Biodiversity Conservation & Research (ABC), Balasore, India; ^9^School of Health Sciences, Health Campus, Universiti Sains Malaysia, Kubang Kerian, Kelantan, Malaysia; ^10^Department of Agricultural Science, Faculty of Agro-Based Industry, Universiti Malaysia Kelantan, Kota Bharu, Kelantan, Malaysia; ^11^Department of Orthopaedics, School of Medical Sciences, Universiti Sains Malaysia, Kubang Kerian, Kelantan, Malaysia

**Keywords:** antibiofilm, dental biofilm, *Clitoria ternatea*, response surface methodology, artificial neural network

## Abstract

The development of biofilm on the biotic and abiotic surfaces is the greatest challenge for health care sectors. At present times, oral infection is a common concern among people with an unhealthy lifestyle and most of these biofilms-associated infections are resistant to antibiotics. This has increased a search for the development of alternate therapeutics for eradicating biofilm-associated infection. Nanobiotechnology being an effective way to combat such oral infections may encourage the use of herbal compounds, such as bio-reducing and capping agents. Green-synthesis of ZnO nanoparticles (ZnO NP) by the use of the floral extract of *Clitoria ternatea*, a traditionally used medicinal plant, showed stability for a longer period of time. The NPs as depicted by the TEM image with a size of 10 nm showed excitation spectra at 360 nm and were found to remain stable for a considerable period of time. It was observed that the NPs were effective in the eradication of the oral biofilm formed by the major tooth attacking bacterial strains namely *Porphyromonsas gingivalis* and *Alcaligenes faecalis*, by bringing a considerable reduction in the extracellular polymeric substances (EPS). It was observed that the viability of the *Porphyromonsas gingivalis* and *Alcaligenes faecalis* was reduced by NP treatment to 87.89 ± 0.25% in comparison to that of amoxicillin. The results went in agreement with the findings of modeling performed by the use of response surface methodology (RSM) and artificial neural network (ANN). The microscopic studies and FT-IR analysis revealed that there was a considerable reduction in the biofilm after NP treatment. The *in silico* studies further confirmed that the ZnO NPs showed considerable interactions with the biofilm-forming proteins. Hence, this study showed that ZnO NPs derived from *Clitoria ternatea* can be used as an effective alternative therapeutic for the treatment of biofilm associated oral infection.

## Introduction

The oral cavity provides an ideal environment for the growth of a large number of microbial communities that are responsible for the development of oral biofilm (Caputo et al., 2018; Morse et al., 2018; Quintieri et al., 2020). The development of oral biofilm takes place under the influence of the salivary glycoproteins, which act as adhering material for the sessile communities (Lahiri et al., 2021a). The sessile communities remain entangled with the help of a self-secreted polymeric substance (EPS) that acts as a natural glue. The EPS not only provides nourishment to the indwelling cells but also prevents the penetration of drug-like molecules resulting in the development of antimicrobial resistance (Khatoon et al., 2018). Thus, the use of alternative therapeutics has become an important way of combating biofilm-associated oral infections. Bio-nanotechnology is an upcoming and booming area in the field of Biotechnology and is coming up as an important part of clinical dentistry and dental practices (Thrall, 2004). Nano-particles are more effective than conventional antimicrobial agents and are used in mouth-wash, toothpaste, mouth-freshener, etc., and also in many oral clinics as a healthcare technology for reducing the risk factor of any kind of surgery (Thrall, 2004). Nano-particle technology or rather bio-nano-particle technology is especially used in the processes of dental filling, enamel polishing for prevention of caries, implantation technology as nano implant particles, which has more effectiveness than the regular type of nanoparticles, etc (Sahoo and Labhasetwar, 2003). A significant part of the nano-strategy lies in their role in the prevention of bacterial growth and bacterial biofilm formation. This nanoparticle technology is getting more and more popular among people as this technology is cost-effective, time-saving, and its application may avoid major surgery (Vasir and Labhasetwar, 2005). Among various types of metallic and non-metallic nanoparticles used, there are many added values of using the zinc oxide nanoparticles as the type selection of the nanoparticle. In comparison to the other types of nanoparticles like silver oxide nanoparticles or copper oxide nanoparticles that are organic or bulk oxide, it is seen that the zinc-oxide nanoparticles are robust, chemically more stable with marked thermal resistance, and have a long shelf life (Vasir et al., 2005). Also, many studies have confirmed that the selective toxicity of ZnO nanoparticles is almost negligible in comparison to the other kinds of nanoparticles, and also while comparing them with other nanoparticles, they show the minimal effect of toxicity on human cells (Maeda et al., 2000). Zinc oxide nanoparticles also show microbicidal properties against Gram-positive and Gram-negative bacteria and fungal spores that are usually resistant to a higher temperature and higher pressure (Allaker, 2011). The advancement of modern nano-strategy due to its effectiveness and preciseness can solve many dental disturbances. The efficiency of nanoparticles can be further increased once they are mixed with some bioactive compounds derived from natural phytoextracts having high medicinal properties and are used traditionally for cleaning and whitening teeth enamel (Allen and Cullis, 2004).

Nanobiotechnology based on a phytocompound-induced synthesis of nanoparticles is becoming a rising tendency in green chemistry due to its simple, non-toxic, and inexpensive nature (Bala et al., 2015). Amongst the preferred nanoparticles, the use of biogenically synthesized ZnO nanoparticles having diverse biomedical applications (Jan et al., 2020) is considered an environment-friendly and convenient technique.

In this study, the flowers of *Clitoria ternatea*, known for their medicinal values, were used as the biogenic source for ZnO nanoparticle production. Traditionally this plant is known mainly for its antistress, antidepressant, and anticonvulsant activities. The ZnO nanoparticles, biogenically formed from this plant extract, are evaluated for their antibiofilm activities against dental biofilm-forming bacterial strains and their superiority will be confirmed over conventional antibiotics. Such appraisal was confirmed by simulation-based studies with some statistical and computational model-based analysis. The *in silico* study helped in understanding the optimum condition for the production of nanoparticles and also helped in understanding the interaction of the ZnO NP with the targeted biofilm-forming proteins.

## Materials and methods

### Microorganism

*Porphyromonsas gingivalis* and *Alcaligenes faecalis* were used in this study. The bacterial cells were cultured in Luria Bertani Broth (LB broth) at 37°C for 24 h.

### Preparation of plant extract

The dried petals of the flower *C. ternatea* were pulverized in water and incubated for a period of 24 h. This was followed by filtering using the gauge filter and the filtrate was stored at 0–4°C for further use (Lahiri et al., 2021c).

### Synthesis of zinc-oxide nanoparticles

Synthesis of ZnO NPs was performed by dissolving 5 mM zinc nitrate in 50 mL Mili Q water and was kept in a stirrer for a period of 1 h (Jamdagni et al., 2018). This was followed by the addition of 25 mL of sodium hydroxide. There was an observed change in the coloration of the solution after incubation for 1 h with plant extract at a volume of 25 mL. The solution was kept under the stirring condition at least for a period of 3 h. The precipitate was separated from the mixture by centrifugation at 8,000 *g* for 15 min. The pellets were dried using a hot air oven at 80°C for 2 h.

## Response surface methodology (RSM) for ZnO NP optimization

The Box–Behnken design (BBD) was considered to model the production of ZnO NP. The pH, volume of extract (mL), reaction time (min), temperature (°C), and concentration of zinc nitrate (mM) were the input parameters, while the absorption characteristics of the synthesized NP were optimized with Design-Expert Version 7.0.0 (Statease Inc; Minneapolis, USA) (Sarkar et al., 2021b).

## Artificial neural network (ANN) teaching-learning based optimization (TLBO) for ZnO NP optimization

The multilayer perception (MLP) neural network along with error backpropagation was considered in this study using MATLAB R2014b (Math Works Inc., USA) (Sarkar et al., 2020; Lahiri et al., 2021c). The neural network model was built of 5 (input layer)−3 (hidden layer)−1 (output layer) architecture (**Figure 3**), learning range varied from 0.5 to 0.9. For non-linear activation functions, the Levenberg–Marquardt ANN tool was used, along with the ANN backpropagation technique for feed-forward neural networks.

The environment of classroom learning and improvement of the student's performance has been simulated in the teaching–learning based optimization (TLBO). The input variables, fitness value, and the initial solution have been assumed as the subject, marks obtained by the students, and the size of the student batch, respectively. In this study, the temperature, pH, time, concentration of ZnNO_3_, and volume of the plant extract were considered as the subject. The values of those parameters were assumed by the student. The evaluation criteria to measure the performance of each student was maximization. In the teacher phase, the teaching factor was introduced to improve the performance of the students. Two random students with unequal performance were selected in the learner phase, in this phase higher performance was attained by learning. Learning from their own interactions as well from the teacher was considered as the iteration. The best-performing student was identified in each iteration. The process continued till the attainment of a maximum number of iterations selected or attainment of the constant best value for the last 50 generations (Morse et al., 2018; Stalin et al., 2019). Here, the absorbance value was assumed as the student's performance and maximization of the absorbance value. The best-performing student was selected from the batch of the absorbance value was considered as the TLBO objective. The TLBO parameters considered for the study are provided in Table 1.

**Table 1:** The parameters selected to conduct the TLBO.

**Attributes for TLBO**	**Value/range**
Student	The set of temperature, pH, time, the concentration of ZnNO_3_, and volume of the plant extract.
Size of the student batch	40
Teaching factor	1–2
Stopping criteria	Attainment of the constant best value for the last 50 generations.

**Figure 1:**
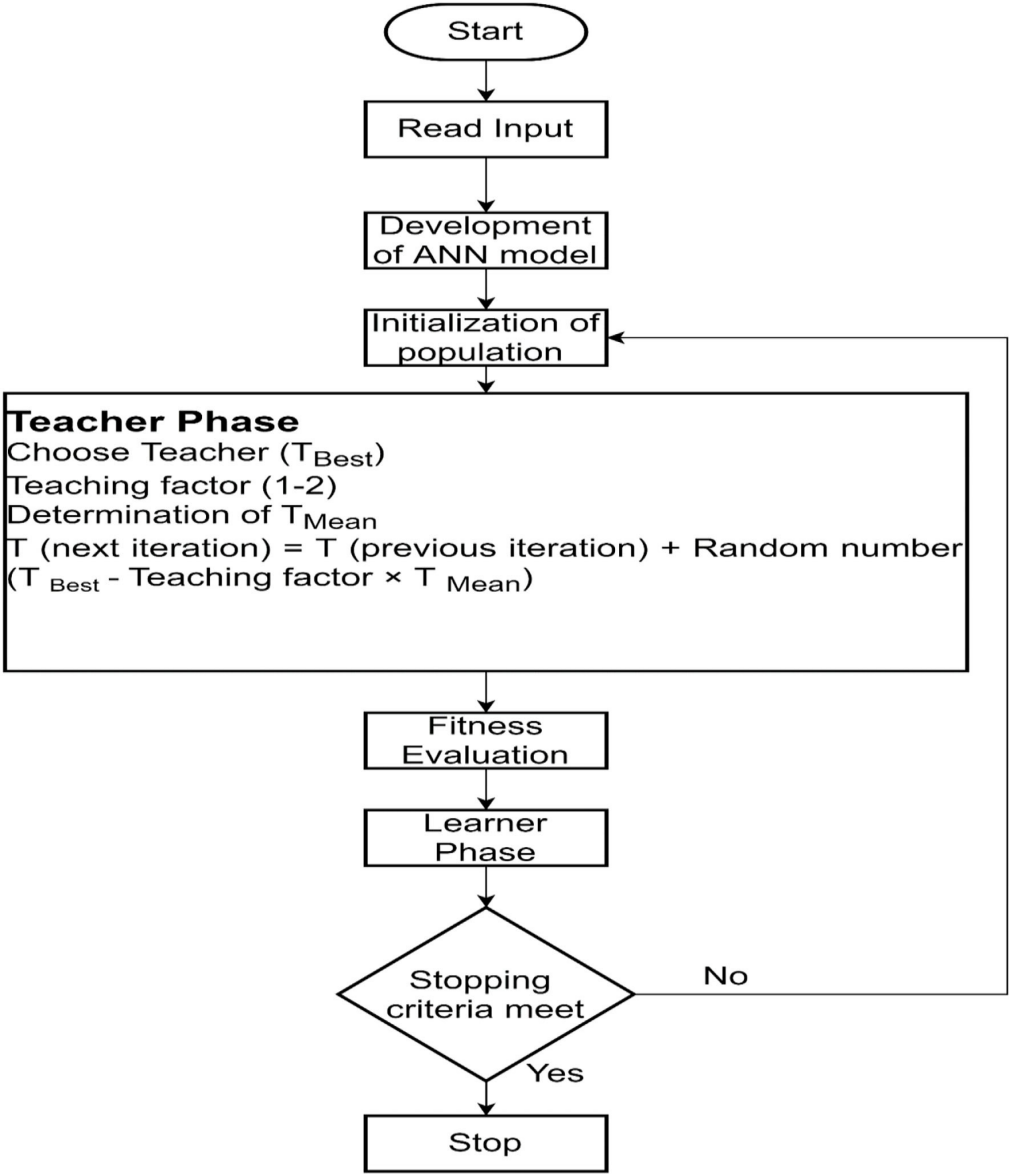
Flowchart for the proposed ANN-TLBO algorithm.

The proposed ANN-TLBO methodology to find the value of the optimum input parameters for maximum absorbance is illustrated in Figure 1. The ANN model was developed with the dataset of the input parameter–response combined with the aim of maximizing the coefficient of correlation (*R*) value. The TLBO was employed considering the size of the student batch of 40, and the student's performance was boosted in the teacher and learner phase till the attainment of the stopping criteria.

### Characterization of the nanoparticles

Aliquots were taken for analyzing the UV–vis spectra at time intervals of 24 h. The UV–vis spectra were analyzed within the range of 200–1,000 nm. Transmission electron microscopy was performed for analyzing the dimension and size of the ZnO NPs (Tailor et al., 2019; Bharadwaj et al., 2021). An X-ray diffractometer is used for the purpose of analyzing crystalline phase and purity between 20° and 80°.

### Biofilm inhibition by ZnO NPs

The antibiofilm effect of the aqueous extract, biogenically synthesized ZnO NPs from *C. ternatea* on *Porphyromonsas gingivalis* and *Alcaligenes faecalis*, was analyzed using the microdilution method (Balouiri et al., 2016). The bacterial cells were grown within the wells of a 96-well plate at a concentration of 1 × 10^6^ CFU/ml followed by incubation at 37°C for a period of 72 h to allow the development of biofilm (Lahiri et al., 2021b,c). After the development of biofilm, it was followed by the addition of ZnO NPs, plant extract, and amoxicillin as standard antibiotics. This was followed by the addition of 3-[4,5-dimethyl-2-thiazolyl]-2, 5-diphenyl-2H-tetrazolium bromide (MTT dye) to quantify the viability of the bacterial cells in the presence of the challenge using the ELISA plate reader (2018 GEN-NET).


(1) $$\begin{array}{l} \text{Percentage Biofilm Inhibition =}\frac{\left[\left(\text{OD of untreated control}\right)\;-\;\left(\text{OD of the treated sample}\right)\right]}{\left(\text{OD of untreated control}\right)}\times 100 \end{array}$$

### Destruction of carbohydrates and proteins associated with biofilm

The density of the sessile population associated with the biofilm is dependent on the total amount of carbohydrates and proteins that are associated with the EPS (Limoli et al., 2015). The process of extracting the carbohydrates and proteins from the biofilm comprises cell incubation in accordance with the biofilm assay. After the incubation period, the biofilm was washed gently using the PBS and boiled for a period of 30 min within 0.5 N NaOH for the purpose of extracting the surface protein. This was followed by centrifugation at 10,000 *g* for a period of 5 min followed by the collection of clear solution. The protein concentration was determined using the Lowry method. The carbohydrate concentration was analyzed by taking the sample of EPS within the test tube followed by the addition of 900 μl of distilled water and 5 mL of 98% of sulphuric acid. The color intensity was analyzed spectrophotometrically at 490 nm.

## Databases and information retrieval

The three-dimensional X-ray crystallographic structures of proteins responsible for biofilm formation, quorum sensing, and motility-related proteins in the EPS, with an approximate resolution of 1.5–2Å, were collected from the Protein Data Bank (https://www.rcsb.org/). The structure of receptor protein PDB ID: 5OLJ is associated with dental biofilm forming bacterial species *Porphyromonas gingivalis*. Another dental biofilm-forming protein was obtained from the isolated bacterial species *Alcaligenes faecalis*, using the phyre2 web server (http://www.sbg.bio.ic.ac.uk/phyre2/) in the.pdb format.

## Determination of the viability count of the sessile group of cells

The working strain grown on 0.1% chitin flakes (w/v) for a period of 72 h was washed with 0.1% (w/v) normal saline to eliminate planktonic groups of cells. After treating the sessile cells with plant extracts and bioactive compounds, the growth was determined spectrophotometrically at 590 nm at varying intervals of time (Baishya et al., 2016).

## ADME evaluation

The prediction of the ADME characteristics of the selected ligands was achieved through the SwissADME web server (http://www.swissadme.ch/). Swiss ADME is an open-source free web tool that computes the drug-likeness of compounds through their physicochemical and pharmacokinetic properties of small molecules (Daina et al., 2017). The input format consisted of the canonical SMILES of the selected compounds and the output from SwissADME containing the descriptors was obtained in.csv file format.

## Nanoparticle drawing with VESTA

In this study, VESTA (Momma and Izumi, 2008) was used for drawing the three-dimensional structure of Zinc oxide nanoparticles (ZnO) in space-filling representation. Visualization for Electronic Structural Analysis or VESTA is a three-dimensional visualization programme for structural models, volumetric data, such as electron or nuclear densities, and crystal morphologies. The software is managed and controlled by the Windows workstations *via* WPKG. ZnO nanoparticle has the lattice parameters *a* = *b* = 3.24940 and c = 5.20380 with alpha = beta = 90 and gamma = 120. Atomic position of Zn in unit cell is [0.33333, 0.66667, 0.00000] and O in unit cell situated at [0.33333, 0.66667, 0.38210].

## Preparation of molecules

The macromolecule preparation for the pre-processing of proteins before docking was achieved by AutoDockTools (ADT), which is a part of MGLTools, from the Molecular Graphics Laboratory at The Scripps Research Institute (Morris et al., 2008). Using ADT, the bound ligands were manually visualized using the Python Molecule Viewer (PMV). Removal of water molecules and addition of hydrogen were also carried out using the same. Finally, Gasteiger charges were added to the cleaned-up protein. Energy minimization of the ligands was achieved using the Auto Optimize tool of the Avogadro molecule editor and visualizer (Hanwell et al., 2012). Auto optimization continuously optimizes molecular geometry through molecular mechanics (Hanwell et al., 2012). The UFF or Universal Force Field was used along with a default value of 4 for “Steps per Update” and the energy minimization was achieved with a dE value of 0. The optimized ligands were written in.pdb format and were subjected to automatic preparation by AutoDock Tools. ADT checked for and merged non-polar hydrogens with the heavier atoms to which they are attached and added Gasteiger charges.

## Grid preparation and molecular docking

To understand how the nanoparticles affected the interactions between the biofilm-associated proteins and the phytocompounds, a double-docking approach was followed, wherein, docking was first performed between the protein and ligand, and the resultant protein–ligand complex with the best cluster and lowest energy rank was further re-docked with zinc-oxide nanoparticles (ZnO-NPs). The whole procedure was carried out using the AutoDockTools software package (Morris et al., 2009). AutoDock is a molecular modeling simulation software specialized for effective protein–ligand docking, which is available under the GNU General Public License and is one of the most cited software used for docking applications in the research community.

AutoGrid was used to pre-calculate the three-dimensional grid of interaction energy based on macromolecular coordinates. AutoGrid constructs a three-dimensional grid surrounding the coordinates for the protein target and calculates the interaction energy of each grid point within it, thus creating a three-dimensional “array” of interaction energies called a “grid map” (Morris et al., 2008). This is done for the rapid evaluation of interaction energies as the completed grid of energies provided a quick lookup table (Goodsell et al., 1996). For both the docking studies, the entire macromolecule or the protein complex was enclosed within the grid box for the programme to create a larger amount of grid maps and search for the best interaction residues instead of limiting it to selective space.

The actual docking simulation was achieved with AutoDock4 for both rigid docking of protein and phytochemical ligand, and the resultant complex with the nanoparticle. A total of 1,000 runs of genetic algorithm (GA) with default parameters were used as search parameters and the output was set to the Lamarckian genetic algorithm (LGA) docking, also known as a hybrid genetic algorithm-local search (GA-LS).

## Attenuation of biofilm by atomic force microscope (AFM)

The biofilm was developed on the surface of the glass slip and was rinsed with phosphate buffer saline maintained at pH 7.4. Green synthesized ZnO NP was used for treating the biofilm being casted on the surface of the glass slide. Both treated and untreated samples of biofilm were scanned by the use of AFM (NT-MDT, Russia) at a speed of 1 Hz.

## Results and discussions

### Optimization in the production of biogenic ZnO NP

**Figure 2:**
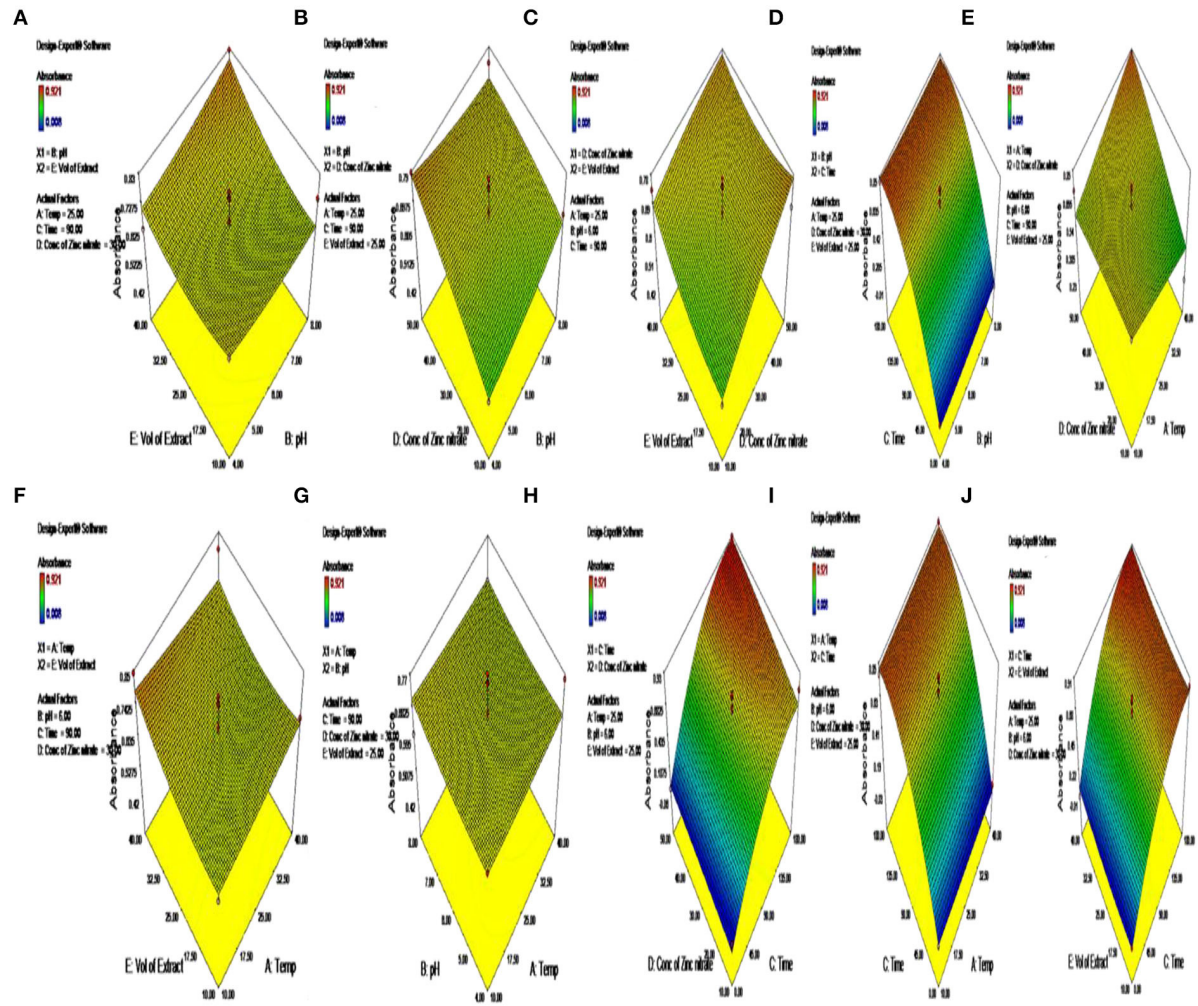
**(A–J)** Optimization of parameters for the synthesis of green-synthesized ZnO by *C. ternatea*.

The parameters that were used for the purpose of optimizing the synthesis of ZnO NPs include pH, the volume of the extract of the plant used, and the concentration of zinc nitrate, which plays an essential role in the mechanism of synthesizing ZnO NPs. Thus, these three important parameters were taken into consideration for further study with RSM. RSM was used to optimize the use of three operational variables: pH, the volume of the extract, and the concentration of zinc nitrate for the purpose of better production of the ZnO NPs (Table 2). The values at *Y* showed that the parameters had a significant effect on the production of ZnO NPs (Table 3). The Box-Behnken design was used for the purpose of optimizing the three variables comprising 46 runs with five replicates of the central point (Figure 2). The optimization of the parameters was conducted one-factor-at-time and the results were in ignorance of the interactions between the process variables. The model helped in predicting the maximum absorbance of the biogenic ZnO NPs that showed an F-value of 15.97 and a *p*-value of <0.01 that greatly implies that the model was used for the purpose of optimizing the conditions of NPs synthesis is significant (Figure 2, Table 2).

**Table 2:** Process parameters for production of biogenic ZnO NP as per Box-Behnken design.

**Run**	**Temp (°C)**	**pH**	**Time (min)**	**Conc of Zinc nitrate (mM)**	**Vol of Extract (mL)**	**Absorbance**
1	25	8	90	50	25	0.742
2	40	6	90	30	10	0.698
3	10	6	180	30	25	0.778
4	40	6	90	50	25	0.784
5	25	4	0	30	25	0.008
6	25	8	180	30	25	0.756
7	25	4	90	30	40	0.638
8	10	8	90	30	25	0.612
9	10	6	90	50	25	0.743
10	40	6	90	30	40	0.796
11	40	6	180	30	25	0.843
12	25	8	0	30	25	0.01
13	25	4	90	50	25	0.789
14	25	8	90	30	40	0.824
15	40	4	90	30	25	0.756
16	25	6	90	30	25	0.425
17	25	6	0	30	10	0.009
18	25	6	0	50	25	0.012
19	25	6	180	50	25	0.921
20	40	8	90	30	25	0.655
21	25	6	90	10	10	0.512
22	25	6	90	30	25	0.768
23	10	6	90	30	10	0.626
24	10	4	90	30	25	0.678
25	25	6	90	50	10	0.684
26	10	6	90	10	25	0.712
27	25	6	180	10	25	0.801
28	25	4	180	30	25	0.814
29	10	6	90	30	40	0.842
30	25	6	0	10	25	0.012
31	10	6	0	30	25	0.014
32	40	6	90	10	25	0.234
33	25	6	90	50	40	0.744
34	25	4	90	10	25	0.521
35	25	6	90	30	25	0.667
36	25	4	90	30	10	0.7
37	25	6	90	10	40	0.734
38	25	6	90	30	25	0.746
39	25	6	90	30	25	0.742
40	25	8	90	30	10	0.745
41	25	6	0	30	40	0.012
42	25	6	180	30	40	0.845
43	25	6	180	30	10	0.848
44	40	6	0	30	25	0.018
45	25	8	90	10	25	0.661
46	25	6	90	30	25	0.621

**Table 3:** RSM optimization model for optimizing the green synthesis of ZnO NPs from *C. ternatea*.

**Nanoparticle**	**Types of Response**	**Response**	**R^2^ value**	**Adjusted R^2^ value**
Green-synthesized ZnO NP from *C. ternatea*	Absorbance = Absorbance	Absorbance	0.9723	0.8755
	+0.12747		
	−0.010575 * Temp		
	−0.015677 * pH		
	+9.91644E-003 * Time		
	+3.74688E-003 * Conc of Zinc		
	Nitrate		
	−6.42454E-003 * Vol of Extract		
	−2.91667E-004 * Temp * pH		
	+1.12963E-005 * Temp * Time		
	+4.32500E-004 * Temp * Conc of		
	Zinc nitrate		
	−1.31111E-004 * Temp * Vol of		
	Extract		
	−8.33333E-005 * pH * Time		
	−1.16875E-003 * pH * Conc of Zinc		
	Nitrate		
	+1.17500E-003 * pH * Vol of Extract		
	+1.66667E-005 * Time * Conc of		
	Zinc nitrate		
	−1.11111E-006 * Time * Vol of		
	Extract		
	−1.35000E-004 * Conc of Zinc		
	Nitrate * Vol of Extract		
	+1.37963E-005 * Temp^2^		
	+3.27604E-003 * pH^2^		
	−3.13863E-005 * Time^2^		
	−3.03646E-005 * Conc of Zinc		
	Nitrate ^2^		
	+1.87130E-004 * Vol of Extract^2^		

### ANN-TLBO prediction

**Figure 3:**
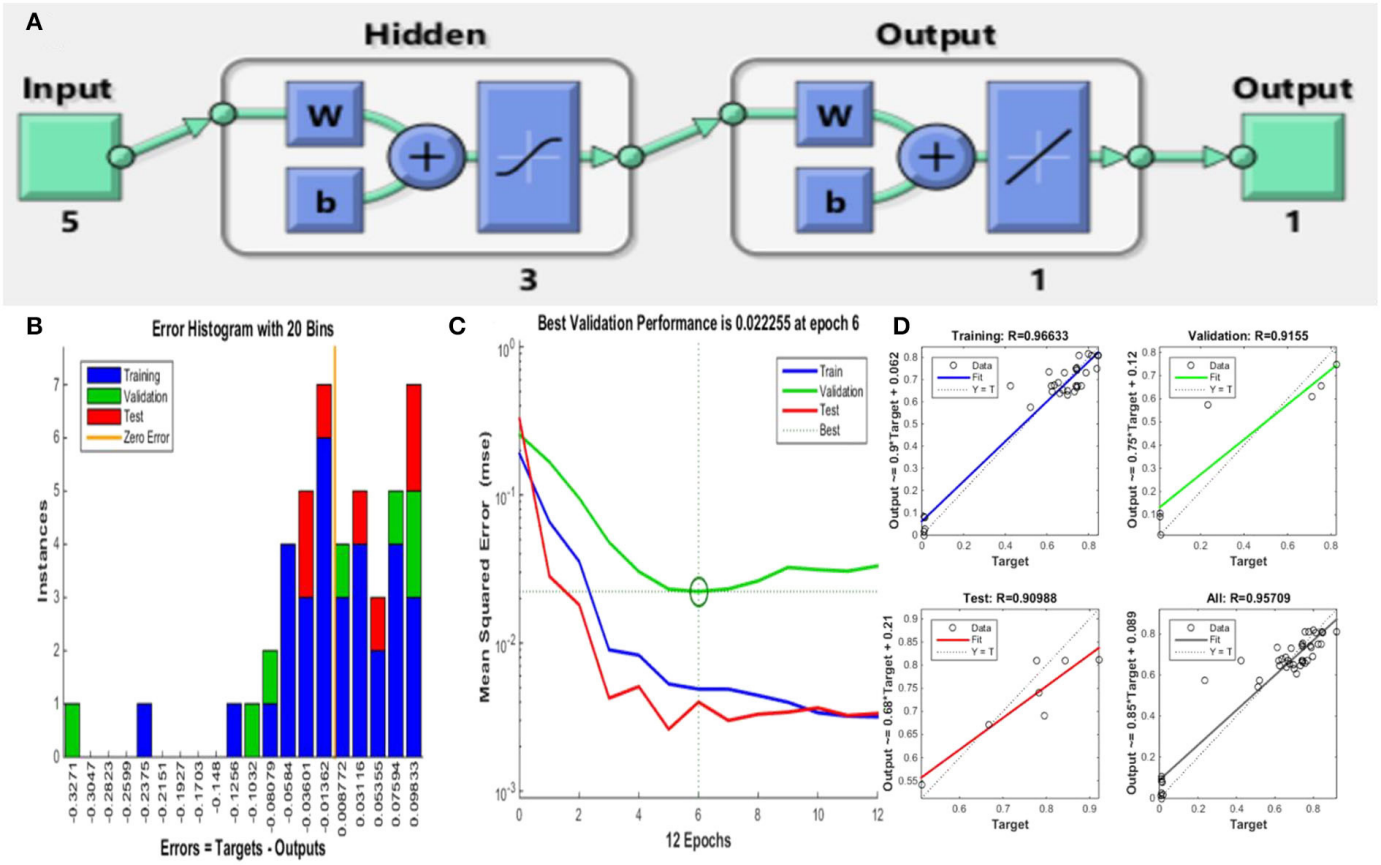
ANN-TLBO approach for ZnO NP synthesis. **(A)** Model architecture, **(B)** error histogram of the proposed model, **(C)** performance plot of the proposed model, and **(D)** regression plot for training, testing, and validation.

The training of the neural network by a subset of various data matrices along with validation and testing regulates the performance of ANN-TLBO (training: testing: validation = 70:15:15). Thus, the ANN-TLBO model is implemented on the investigational data obtained from the executed BBD, the data set was split into three subsets (32:7:7) for the purpose of training, testing and validating the model. The mean square obtained from the analysis is considered as an indicator of performance, and *R*^2^ (Correlation coefficient) is considered the precision index (Sarkar et al., 2020, 2022). The regression plots of the neural networks help in the representation of the training set, testing set, and validation set. The ANN-TLBO model for determining the biogenic ZnO nanoparticle synthesis comprises five input neuron layer that represents the process parameters, three hidden, and one output layer with the correlation coefficient value of 0.99939. On the other hand, *Staphylococcus aureus* comprises one input and four hidden neuron layers with a correlation coefficient value of 0.95709. It was further observed that the predicted values by ANN-TLBO were in close proximity with the actual run and the higher *R*^2^ value in comparison to that of the RSM model illustrated that it was able to build a more robust model in comparison to that of the RSM technique (Figure 3, Table 3).

## Characterization of the green synthesized ZnO NPs

### Characterization of green synthesized ZnO NP

**Figure 4:**
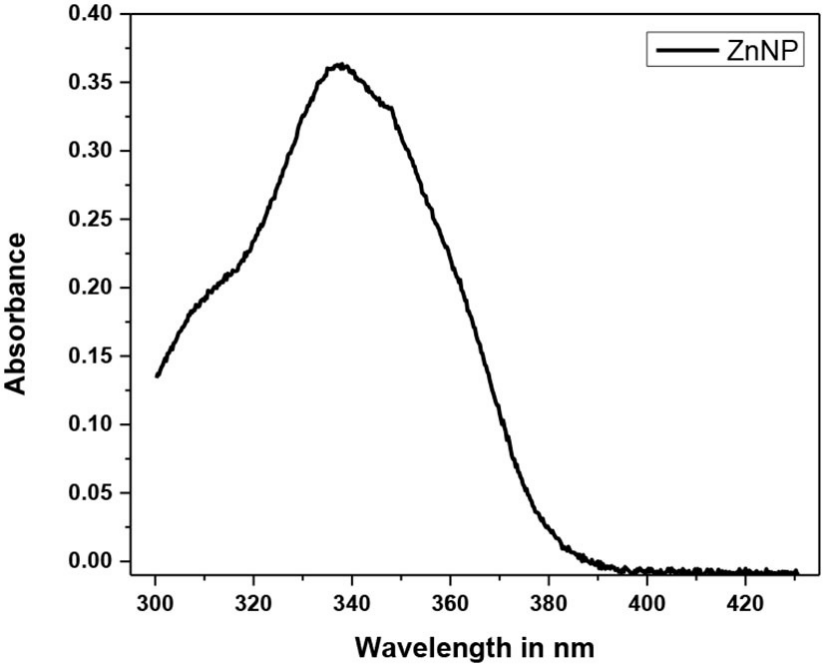
UV–visible spectroscopic analysis Green synthesized ZnO NP.

**Figure 5:**
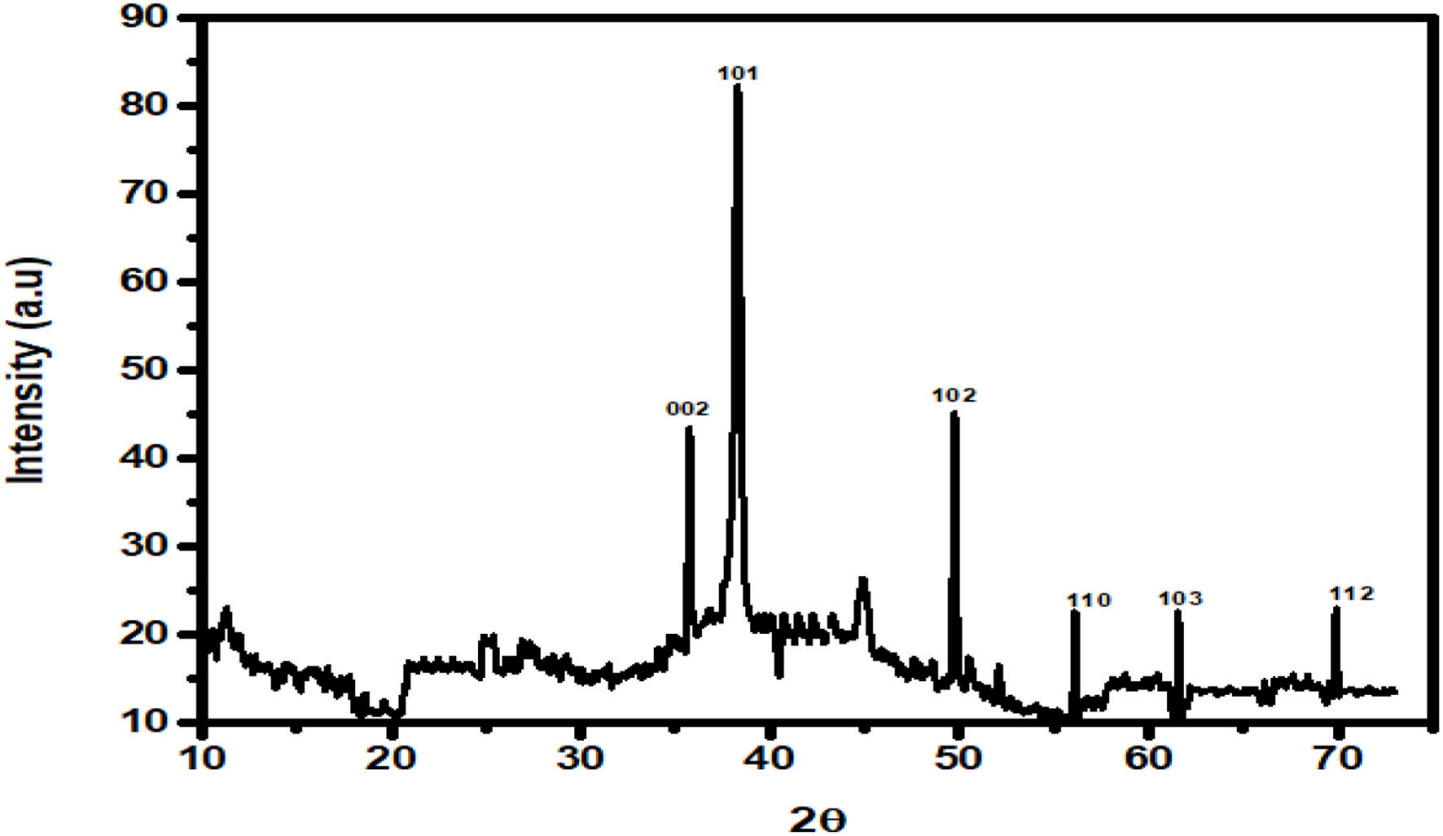
XRD analysis of the green synthesized ZnO NPs.

The green synthesis of ZnO nanoparticles from *C. ternatea* extract includes plant secondary metabolites those act as reducing as well as stabilizing agents of Zn ions in solutions of zinc oxide. The green synthesis of ZnO NPs from *C. ternatea* showed a broad peak at 335 nm (Figure 4) when scanned in the wavelength range of 300–450 nm. For ZnO nanoparticles, the absorbance peak is reported between 310 and 360 nm of wavelength (Song and Yang, 2016; Jayachandran and Nair, 2021) (Figure 4). The XRD patterns obtained by XRD (Figure 5) showed the synthesis of ZnO NP. The pattern of the crystalline peaks corresponding to (002), (101), (102), (110), (103), and (112) was almost similar to the work performed by previously published work (Alahmdi, 2022).

**Figure 6:**
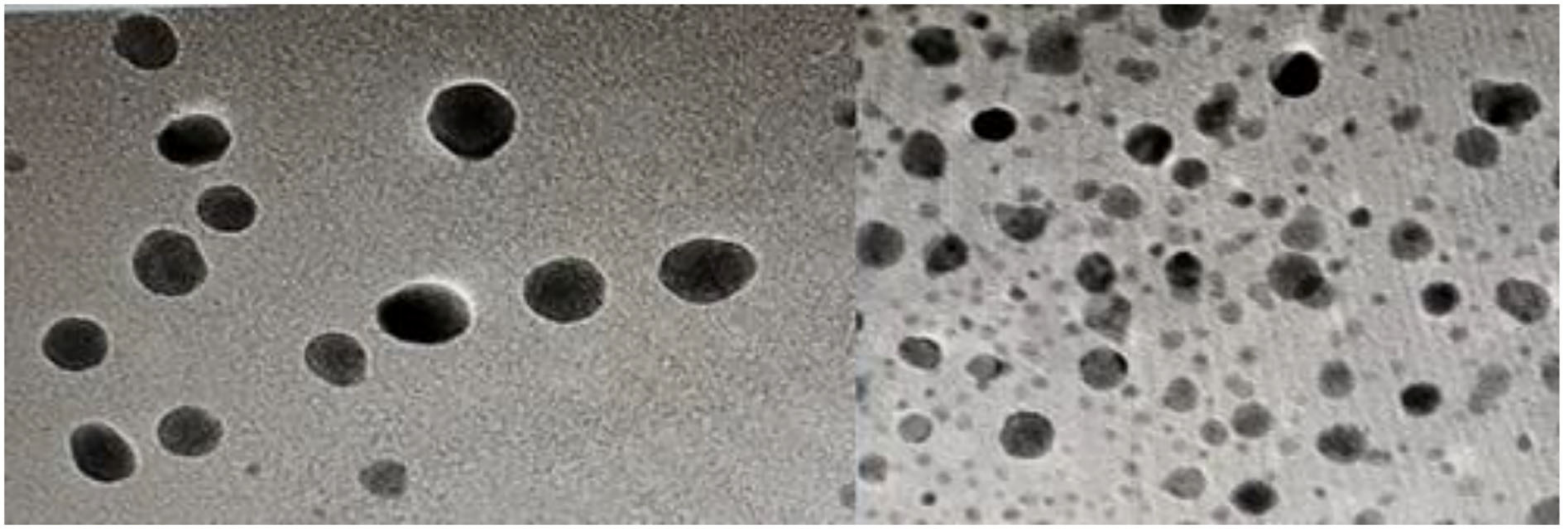
TEM images of green-synthesized ZnO NP from *C. ternatea*.

The green synthesized ZnO NPs thus formed were observed to be spherical in shape within the size range of 10–20 nm when observed with TEM (Figure 6).

### Enfeeblement of the biofilm

**Figure 7:**
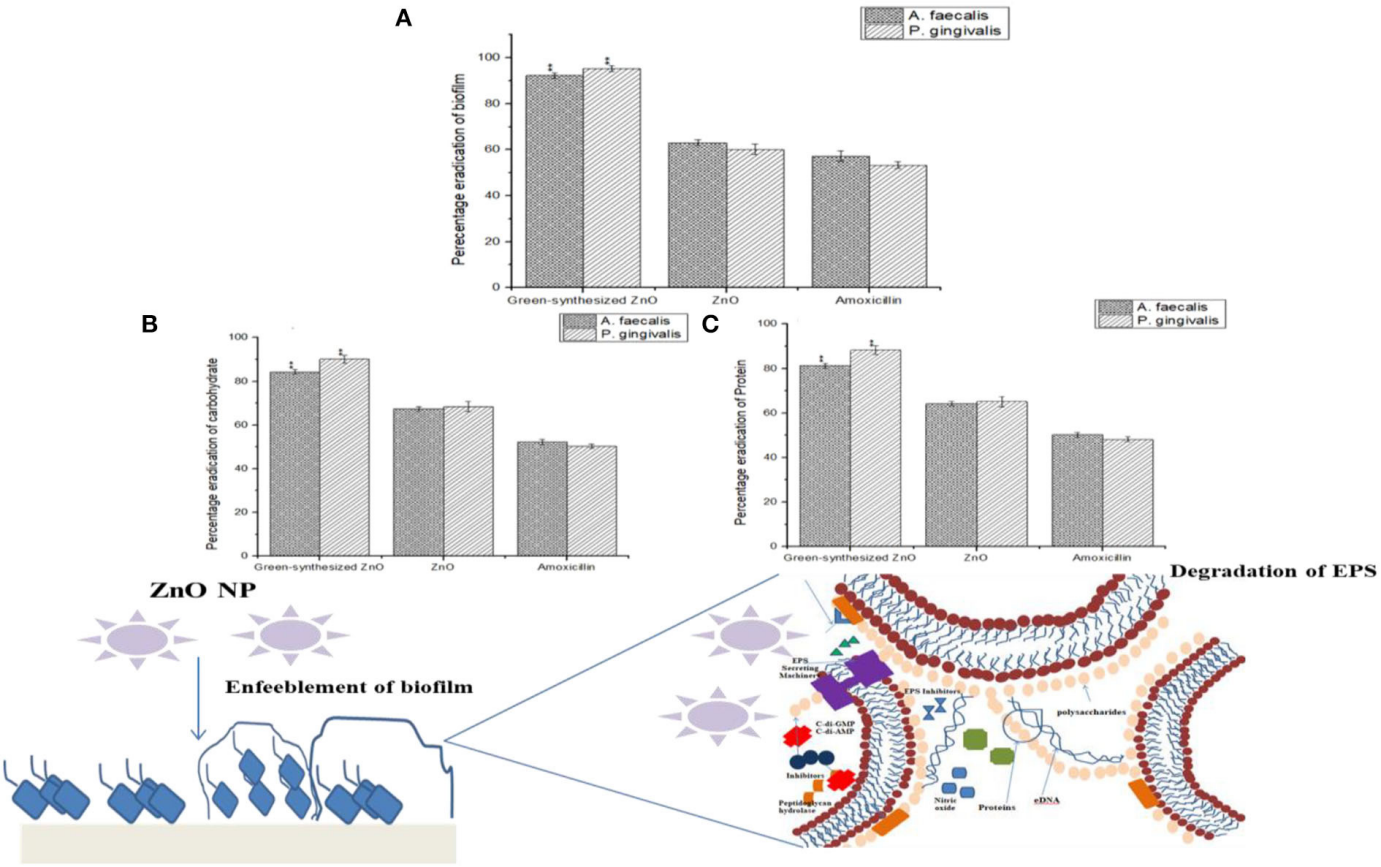
**(A)** Reduction of biofilm formed by *A. faecalis* and *P. gingivalis* in the presence of green-synthesized ZnO NPs. The ZnO NPs were also responsible for a marked reduction in the EPS with the degradation of **(B)** the carbohydrate and **(C)** protein content.

The green-synthesized ZnO NPs were able to bring about the degradation of the biofilm with the enfeeblement of the structural components of the EPS constituting the biofilm (Hsueh et al., 2015). It was observed that the green-synthesized ZnO NPs were able to reduce the biofilm formed by *A. faecalis* and *P. gingivalis* by 92.27 ± 1.22% and 95.27 ± 1.28%, respectively. This was similar to the work done by Husain et al. (2022), which showed that the biosynthesized ZnO NPs were able to bring about degradation of the biofilm formed by *E. coli, S. aureus*, and *P. aeruginosa* (Husain et al., 2022). The destabilization of biofilm architecture can be possible with the degradation of the EPS matrix (Pinto et al., 2020). The EPS has two architectural components, carbohydrates and proteins, which provide strength to the biofilm structure (Flemming et al., 2007). It was observed that the green-synthesized ZnO NPs were able to bring a marked reduction in the carbohydrate and protein content of the EPS. It was observed that the carbohydrate content within the EPS of *A. faecalis* and *P. gingivalis* was markedly reduced by 84.26 ± 1.09 % and 90.12 ± 1.09%, respectively, on the action of green-synthesized ZnO NPs. The NPs were also responsible for a marked reduction in the protein content of the EPS by 81.26 ± 1.09% and 88.23 ± 1.89%, respectively. This observation greatly portrayed the biogenic ZnO NPs were effective in the enfeeblement of biofilm by bringing about the destruction of the EPS (Lahiri et al., 2021d) (Figure 7).

### Degradation of genomic DNA and RNA content

**Figure 8:**
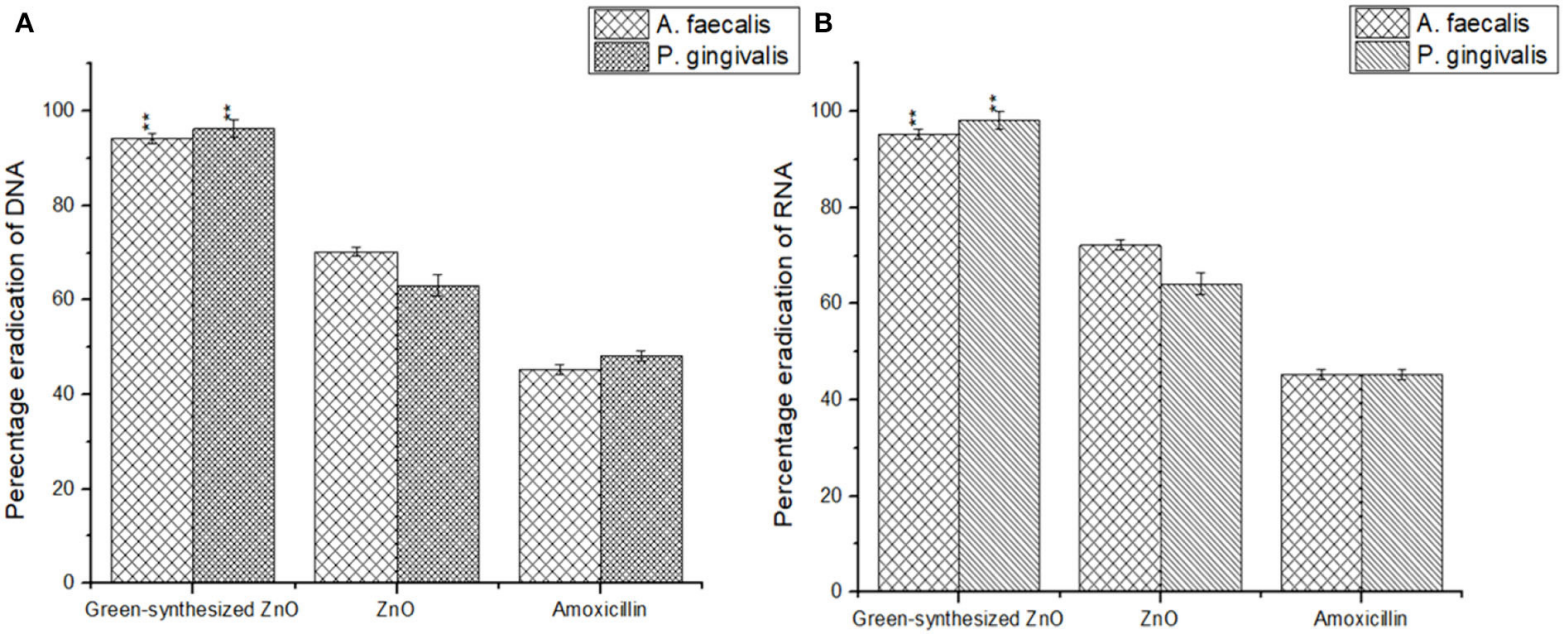
Biogenic ZnO NPs were responsible for a marked reduction in the DNA **(A)** and RNA **(B)** content of the sessile microbial colonies of *A. faecalis* and *P. gingivalis*.

The biogenic ZnO NPs possess the ability to penetrate through the biofilm and act on the cells by bringing degradation in the genomic DNA and RNA content of the cell (Kamli et al., 2021). The biogenic ZnO NPs brought a marked reduction in the genomic DNA content of *A. faecalis* and *P. gingivalis* by 94.26 ± 1.09% and 96.23 ± 1.89%, respectively. It was also observed that the NPs were further responsible to reduce the RNA content of the sessile cells in both *A. faecalis* and *P. gingivalis* by 95.26 ± 1.01% and 98.23 ± 1.83%, respectively (Figure 8).

## Influence on sessile cell viability in the presence of ZnO NP

### Disruption of biofilm under static conditions: Microscopic studies

#### SEM analysis

**Figure 9:**
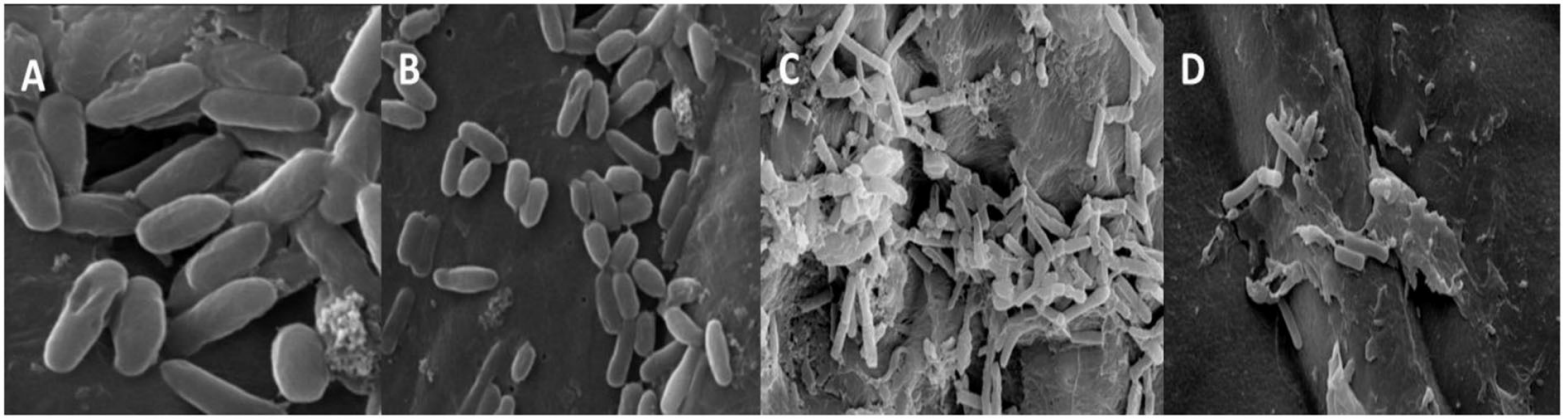
SEM images of *P. gingivalis* **(A,B)** control and treated with biogenic ZnO NPs and *A. faecalis* control and treated **(C,D)** with biogenic ZnO NPs.

The morphological and numerical alterations of biofilm-producing cells of the *A. faecalis* and *P. gingivalis* imparted by the green-synthesized ZnO NP were reflected by the scanning electron micrographs (Figure 9). The ZnO NP brought about a significant reduction in the number of biofilm-producing cells but also brought about a notable shrinkage in the cellular morphology and fewer colonization areas were visible compared to the control. Bacterial cells were found to form different layers of extracellular polymeric substance (EPS) in the control set which upon treatment with ZnO NP was reduced to single layers of cells showing a visible loss of the EPS and release of the cytoplasmic content. This shows that the NP brings about a substantial reduction in the biofilm along with considerable elimination of the sessile microcolonies.

#### Fluorescence microscopic analysis

**Figure 10:**
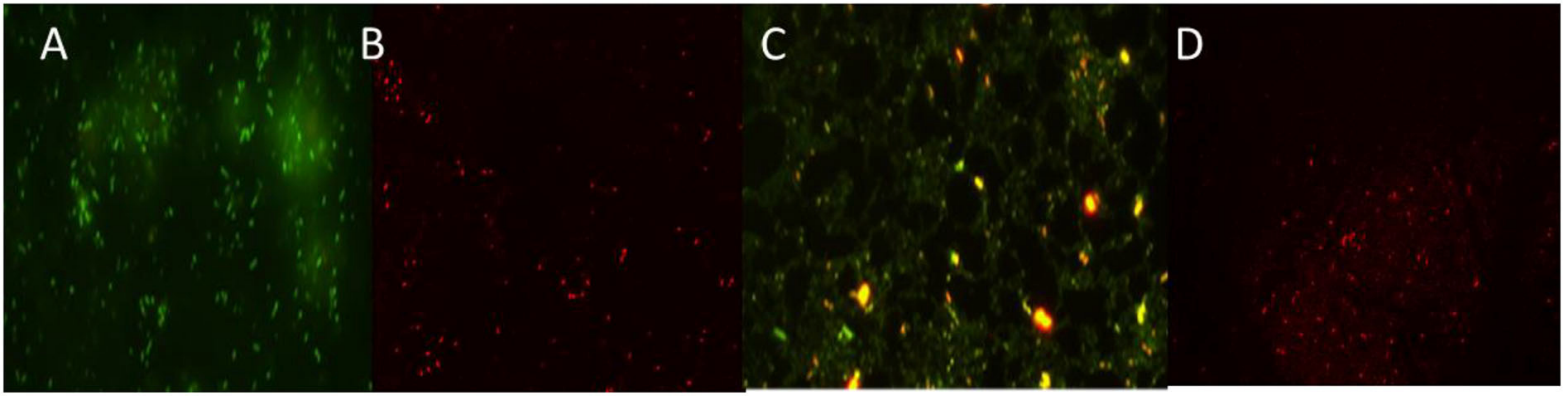
Fluorescence images of untreated and treated sessile cells by ZnO NPs of *P. gingivalis* **(A,B)** and *A. faecalis* **(C,D)**.

The loss of biofilm production ability by the green synthesized ZnO-challenged cells of *A. faecalis* and *P. gingivalis* was checked by the fluorescence microscopic study (Figure 10). It showed a thick coating of biofilm in untreated conditions, whereas the treatment with ZnO NP resulted in a scattered appearance with a much lesser number of cells and a significantly reduced amount of biofilm. The application of the ZnO NP resulted in the killing of the cells as they appeared reddish in color in the presence of propidium iodide.

#### ADME analysis

In this study, all the selected ligands were observed to have <10 hydrogen donors; however, azadirachtin was observed to have 16 hydrogen acceptors that resulted in a Lipinski violation. Apart from that, azadirachtin also had a molecular weight >500. Except for azadirachtin and nimbin, all the compounds had zero violations of Lipinski's rule of drug-likeness. However, azadirachtin was found to have the highest synthetic accessibility. All the compounds except the same had high gastrointestinal absorption, and none of the compounds are permeable to the blood brain barrier (Table 4).

**Table 4:** Drug-like properties of phytocompounds.

**Bioactive compounds**	**Structure**	**Mol. Wt.**	**H-bond acceptors**	**H-bond donors**	**TPSA**	**Rotatable bonds**	**Bio-availability Score**	**Lipinski violations**
Azadirachtin	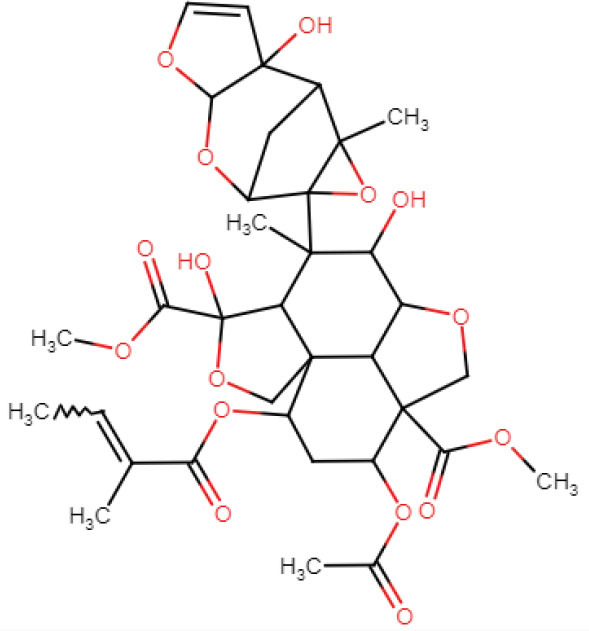	720.71	16	3	215.34	10	0.17	2
Quercetin	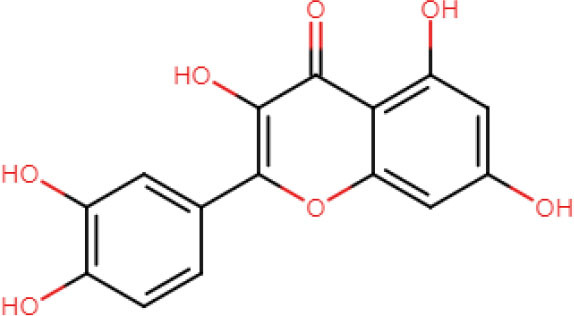	302.24	7	5	131.36	1	0.55	0

#### Docking analysis

AutoDock uses a “hybrid” force field that employs a “full” desolvation model, and also considers directionality in hydrogen bonds (Sarkar et al., 2021a). To estimate the interaction energy, it calculates the energy of ligand and protein in the unbound state first and then calculates the energy of the protein-ligand complex. Finally, these values are collectively used to compute the lowest and mean binding energy values. The lowest the energy value, the better is the interaction score of docking. In this study, nimbolide was found to have the best interaction with both 5OLJ and DMR19 proteins. Each of the compounds is observed to have inhibition constants in the micromolar range (uM). The inhibition concentration is a measure of the potency of an inhibitor. Mathematically, it is the concentration required to produce half the maximum inhibition.

**Figure 11:**
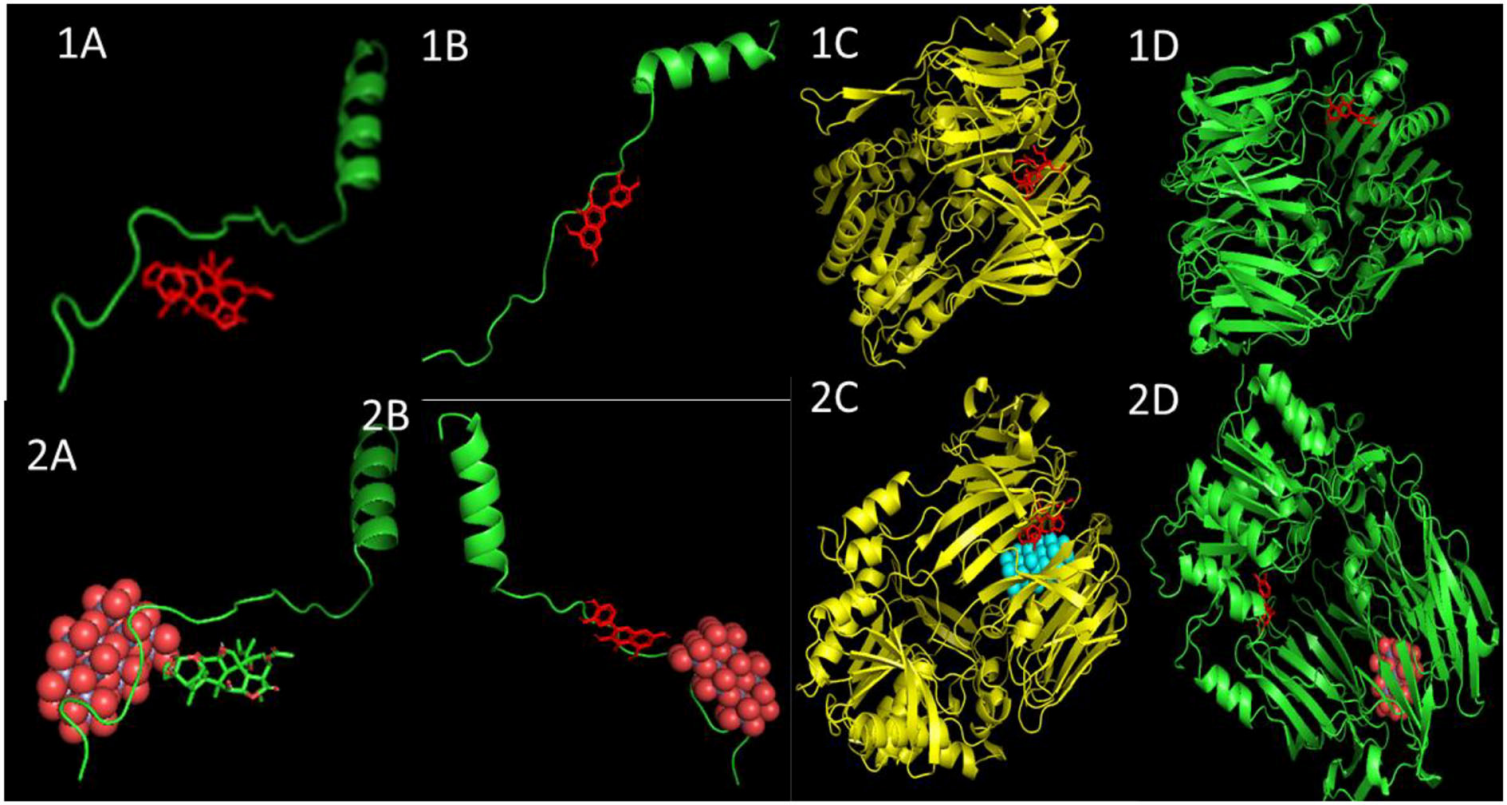
Docked poses and three-dimensional interactions of biofilm producing bacterial protein *P. gingivalis* and *A. faecalis* with phytochemical: **(1A,C)** Azadirachtin, **(1B,D)** Quercetin and the respective phytocompounds along with nanoparticles **(2A,C)** and **(2B,D)**.

The binding energy values of all complexes significantly decreased by ~3 units. Double-docking with the ZnO nanoparticle was also found to enhance the number of cluster formations in all the complexes. In nimbolide, the number of clusters was found to be enhanced by a factor of 21 on docking with nanoparticles. ZnO nanoparticles also had a considerably lower inhibition constant (Ki), which was in the picoMolar and nanoMolar concentrations for biofilm forming proteins *P. gingivalis* and *A. faecalis*, respectively. This implies that a very low concentration of ZnO nanoparticles can be used to get the desired strong inhibition of the biofilm formation (Figure 11, Table 5).

**Table 5:** The binding energy values of molecular docking interaction.

**Protein**	**Ligand**	**Lowest binding energy**	**Lowest binding energy (NP)**	**Mean binding energy**	**Mean binding energy (NP)**	**Inhibition Constant Ki (ligand)**	**Inhibition Constant Ki (NP)**
*P. gingivalis*	Azadirachtin	−8.12	−13.19	−8.11	−12.76	1.12 uM	216.08 pM
	Quercetin	−7.54	−12.31	−7.09	−12.08	2.95 uM	953.67 pM
*A. faecalis*	Azadirachtin	−7.89	−9.77	−7.87	−9.74	8.96 uM	159.60 nM
	Quercetin	−5.80	−8.59	−5.77	−8.46	55.94 uM	506.93 nM

## Detection of attenuation of biofilm by atomic force microscope (AFM)

**Figure 12:**
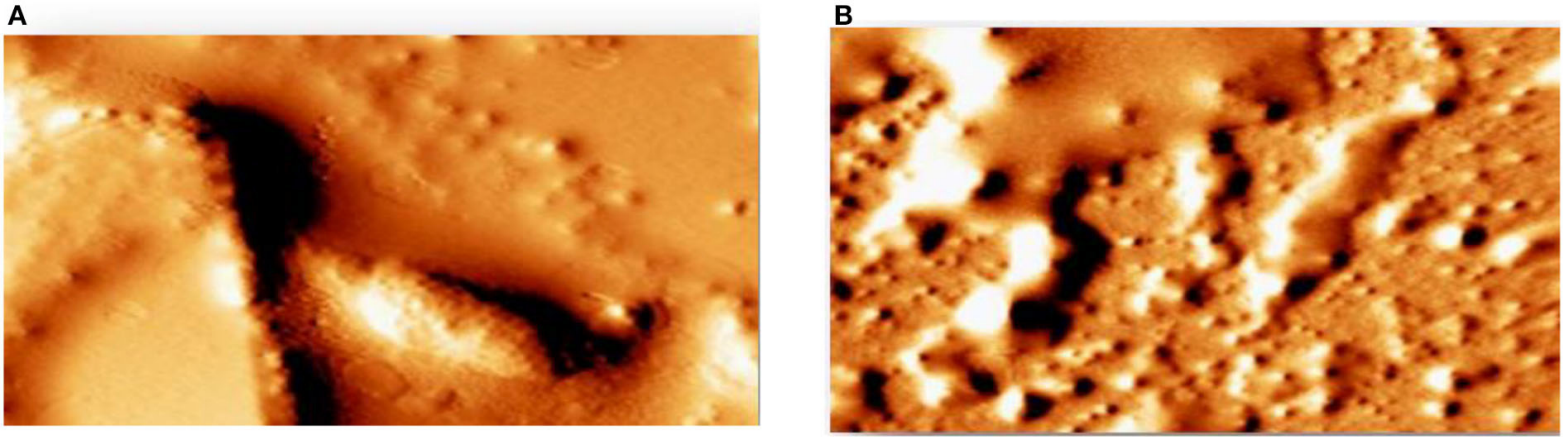
Detection of attenuation of biofilm by Atomic force microscope **(A)** tested strains was able to form a biofilm **(B)** green synthesized ZnO NP decreased significantly the adherence to glass.

AFM revealed that the tested strains was able to form biofilm and had the potency to adhere to a glass surface (Figure 12A). On the other hand, green synthesized ZnO NP decreased significantly the adherence to glass (Figure 12B). In addition, the combination of green synthesized ZnO NP decreased significantly the biofilm formation. AFM clearly showed the disrupted surface topology and height distribution profile of the biofilm developed in the presence of NP compared to the control biofilm.

## Conclusion

This study is focused on the efficiency of the green-synthesized ZnO NPs in the elimination of biofilm formed by *A. faecalis* and *P. gingivalis*. This was further ratified by the *in silico* studies. The recent study focused on the bioaugmented production of ZnO-NPs using floral extract of *C. ternatea* revealed the fact that it has a great possibility for making a nano-based herbal mouthwash with antibiofilm activities, which may be used for checking oral biofilm-induced diseases like periodontitis and tooth loss.

## Data availability statement

The original contributions presented in the study are included in the article/supplementary material, further inquiries can be directed to the corresponding author/s.

## Author contributions

Conceptualization and writing—review and editing: DL, MN, SPat, TS, and RR. Methodology: DL, MN, TS, SPan, SG, and MM. Formal analysis: DL, MN, MM, and RR. Investigation: DL, MN, and RR. Writing—original draft preparation: DL, MN, ZA, HE, SPat, TS, and RR. All authors have read and agreed to the published version of the manuscript.

## Conflict of interest

Author SPat was employed by NatNov Bioscience Private Ltd. and VU was employed by AMH Energy Pvt. Ltd. The remaining authors declare that the research was conducted in the absence of any commercial or financial relationships that could be construed as a potential conflict of interest.

## Publisher's note

All claims expressed in this article are solely those of the authors and do not necessarily represent those of their affiliated organizations, or those of the publisher, the editors and the reviewers. Any product that may be evaluated in this article, or claim that may be made by its manufacturer, is not guaranteed or endorsed by the publisher.
